# Uncovering genetic contributors to developmental delay and intellectual disability: a focus on CNVs in pediatric patients

**DOI:** 10.3389/fgene.2025.1539902

**Published:** 2025-06-23

**Authors:** Yilun Tao, Hongzhi Guo, Dong Han, Miao Yang, Ting Lun, Lihong Wang, Wenxia Song, Haiwei Wang, Xiaoze Li

**Affiliations:** ^1^ Medical Genetic Center, Changzhi Maternal and Child Health Care Hospital, Changzhi, Shanxi, China; ^2^ Precision Medicine Research Division, Changzhi Maternal and Child Health Care Hospital, Changzhi, Shanxi, China; ^3^ Department of Child rehabilitation, Changzhi Maternal and Child Health Care Hospital, Changzhi, Shanxi, China; ^4^ Department of Pediatrics, Changzhi Maternal and Child Health Care Hospital, Changzhi, Shanxi, China; ^5^ Science and Education Division, Changzhi Maternal and Child Health Care Hospital, Changzhi, Shanxi, China

**Keywords:** copy number variations, SNP array, WES, developmental delay, intellectual disability

## Abstract

**Background:**

Developmental delay (DD) and intellectual disability (ID) are prevalent in children and often have genetic causes, particularly copy number variations (CNVs). Chromosomal microarray analysis (CMA) and whole-exome sequencing (WES) are key diagnostic tools for identifying genetic contributions to these disorders. This study assesses the prevalence and clinical impact of CNVs in pediatric DD and ID patients.

**Methods:**

Ninety-nine pediatric patients with DD or ID underwent CMA or WES. Of these, 82 received SNP array analysis, while 17 had WES. CNV pathogenicity was assessed using established databases and ACMG guidelines, with inheritance patterns determined where possible.

**Results:**

Across the 99 patients, 43 CNVs were identified in 40 individuals, with 32 classified as clinically significant, resulting in a diagnostic rate of 30.3%. These findings included 24 deletions (75%), 7 duplications (22%), and 1 instance of loss of heterozygosity (3%). Of the CNVs with known inheritance, 65.2% were *de novo*. Recurrent CNVs made up 36.4% of the total, especially in regions 15q11.2-q13.1, 16p11.2, and 22q11.2. Additionally, 11 CNVs were categorized as variants of uncertain significance (VOUS).

**Conclusion:**

This study supports CMA as an effective diagnostic tool for DD and ID, highlighting the importance of family-based CNV testing for genetic counseling. The findings emphasize the need for comprehensive genetic testing to improve diagnostic accuracy, with future multi-omics approaches potentially clarifying VOUS mechanisms and CNV variability in neurodevelopmental disorders.

## 1 Introduction

Developmental delay (DD) and intellectual disability (ID) rank among the most common neurodevelopmental disorders in children, affecting approximately 1%–3% of the global pediatric population (Marrus and Hall, 2017). These conditions often manifest through delays in language acquisition, motor skills, social interactions, and cognitive development, posing significant challenges to affected individuals and their families. Identifying the underlying etiology is essential, not only for accurate diagnosis and prognosis but also for genetic counseling and targeted interventions (Xiang et al., 2021).

Genetic factors are now recognized as significant contributors to DD and ID, with studies indicating a genetic basis in up to 50% of cases (Jansen et al., 2023; Shchubelka et al., 2024). Copy number variations (CNVs)—duplications or deletions of DNA segments—are key abnormalities linked to these disorders. Chromosomal microarray analysis (CMA), a high-resolution diagnostic tool, has become the first-tier method for detecting CNVs in patients with DD, ID, autism spectrum disorders (ASD), and congenital anomalies, achieving a diagnostic yield of 15%–25%, markedly higher than traditional karyotyping, especially for small microdeletions and duplications (Jang et al., 2019; Kamath et al., 2022; Perovic et al., 2022). Studies have shown that pathogenic CNVs are often associated with complex phenotypes, including DD, ID, epilepsy, and dysmorphisms, and that inheritance patterns—whether inherited or de novo—can offer insights into the severity and recurrence risk, with *de novo* CNVs typically indicating more severe clinical presentations. CMA thus serves as a critical tool for identifying causative genomic variations, aiding in clinical management and personalized treatment, especially in unexplained cases of DD and ID (Miclea et al., 2022; João et al., 2024).

This study aims to analyze the prevalence and clinical significance of CNVs in a cohort of pediatric patients with DD and ID using CMA. By examining CNV types, inheritance patterns, and associated phenotypes, this research seeks to expand the understanding of the genetic landscape of DD and ID and reinforce the utility of CMA as a diagnostic tool in neurodevelopmental disorder diagnosis. Furthermore, this study aims to contribute to improved genotype-phenotype correlations, ultimately supporting more informed clinical decision-making and genetic counseling for affected families.

## 2 Materials and methods

### 2.1 Subject

This retrospective descriptive study was conducted at the Changzhi Maternal and Child Health Care Hospital, targeting pediatric patients diagnosed with dd or ID between August 2021 and December 2022. A total of 99 pediatric patients, each evaluated by clinical geneticists, were included. The study protocol received approval from the Institutional Ethics Committee (CZSFYLL 2021-015), and informed consent was obtained from each participant’s parent or guardian for molecular diagnostic testing and research use. Of these patients, 82 underwent single nucleotide polymorphism (SNP) array testing, while 17 were assessed with whole-exome sequencing (WES) due to complex or specific clinical features.

### 2.2 DNA extraction

Peripheral blood samples were collected from each patient. DNA extraction was conducted using the microsample genomic DNA extraction kit (DP316, Tiangen BioTech Co., Ltd., Beijing, China) according to the manufacturer’s protocol. The concentration and purity of the extracted genomic DNA were measured using a NanoDrop 2000 spectrophotometer (Thermo Scientific), and samples were stored at −20°C until further processing.

### 2.3 SNP array analysis

SNP array analysis was performed on 82 patients using the Affymetrix CytoScan^®^ 750K array kit (Affymetrix, Inc., Santa Clara, CA, United States) following the manufacturer’s instructions. Resulting data were processed to detect CNVs using Affymetrix Chromosome Analysis Suite (ChAS) Software version 3.3.

### 2.4 WES analysis

For the 17 patients selected for WES, the analysis utilized a whole-exome capture kit from MyGenostics Inc. (Beijing, China). SNP and InDel detection was conducted following previously established protocols (Tao et al., 2023). CNVs were identified through relative read depth analysis of NGS data at targeted positions, employing CNVkit (https://cnvkit.readthedocs.io/en/stable/), as outlined in prior studies (Nord et al., 2011; Quenez et al., 2021).

### 2.5 Pathogenicity assessment

The pathogenicity of CNVs was evaluated using published literature and several public databases, including DGV (http://dgv.tcag.ca/dgv/app/home), ClinGen (https://www.clinicalgenome.org/), DECIPHER (https://decipher.sanger.ac.uk/), ClinVar (https://www.ncbi.nlm.nih.gov/clinvar/), gnomAD (https://gnomad.broadinstitute.org/) and OMIM (https://www.omim.org/). This analysis followed the guidelines of the American College of Medical Genetics and Genomics (ACMG) and the Clinical Genome Resource (ClinGen), using the 2020 classification standards (Riggs et al., 2020).

## 3 Results

### 3.1 Patient demographics and clinical characteristics

In this study, 99 pediatric patients with a diagnosis of DD or ID were analyzed (Table 1). The cohort included 61 males and 38 females, ranging in age from 2 days to 33 years. The primary clinical features observed were DD in 67 patients and ID in 62 patients. In addition to these primary diagnoses, other frequently observed symptoms included hypotonia (8 cases, 8.08%), delayed or absent speech (12 cases, 12.1%), and abnormal facies (8 cases, 8.08%), which often correlate with genetic syndromic presentations. Autism spectrum-related symptoms were present in 8 cases (8.1%), while seizures were reported in 6 cases (6.1%).

**TABLE 1:** Summary of patient demographics and clinical features.

Demographic and clinical features	n (%)
Gender	99 patients
Male	61 (61.6%)
female	38 (38.4%)
Age	2 days - 33 years
< or = 5 years	77 (77.8%)
>5 years	22 (22.2%)
Clinical features	99 patients
DD	67 (67.7%)
ID	62 (62.6%)
Delayed Speech	12 (12.1%)
Hypotonia	8 (8.1%)
Abnormal facies	8 (8.1%)
Autism traits	8 (8.1%)
Seizures	6 (6.1%)

### 3.2 CNV detection and classification

**FIGURE 1:**
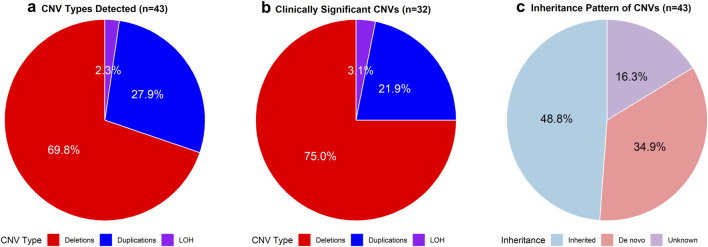
Distribution of CNV types. **(a)** The number and proportion of deletions, duplications, and loss of heterozygosity (LOH) among all detected CNVs (n = 43). **(b)** The number and proportion of deletions, duplications, and LOH among clinically significant CNVs (n = 32). **(c)** Inheritance pattern of all detected CNVs (n = 43).

**FIGURE 2:**
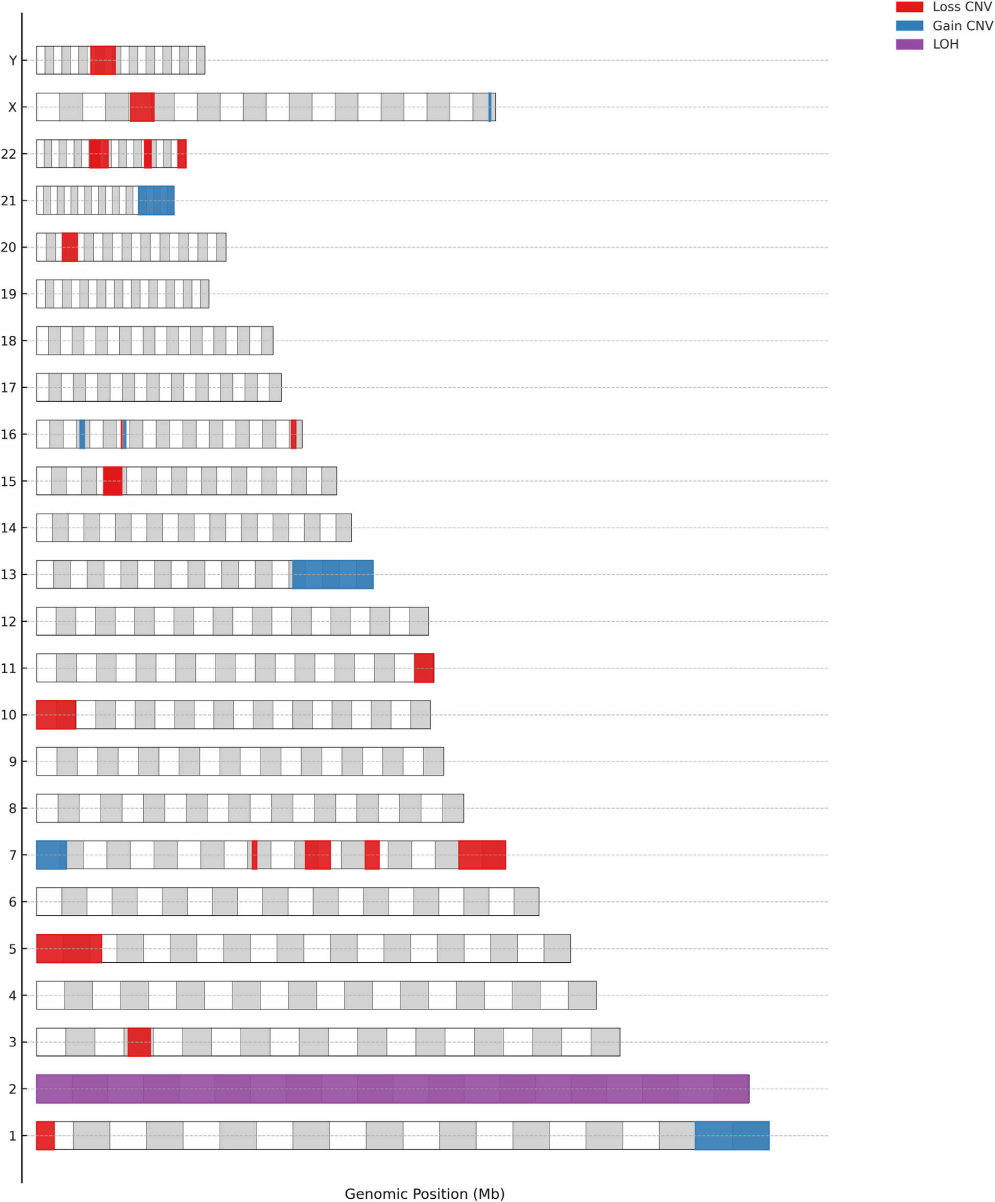
Chromosomal distribution of pathogenic or likely pathogenic CNVs.

In this cohort of 99 pediatric patients, CMA or WES identified 43 CNVs in 40 patients (Figure 1). Among these, 32 CNVs were deemed clinically significant, impacting 30 patients (30.3%); 31 (96.9%) were classified as pathogenic, while 1 (3.1%) was considered likely pathogenic (Table 2; Figure 2). Additionally, 11 CNVs were identified in 11 patients and were classified as variants of uncertain significance (VOUS) (Supplementary Table 1).

**TABLE 2:** Pathogenic or likely pathogenic CNVs observed in DD/ID patients.

No	Age (years)	Gender	CNV position	del/dup	Size	Inheritance status	Classification	Critical region/gene	Clinical symptoms
1	1	M	arr [hg38] 15q11.2q13.1 (23,123,715–28,315,518)×1	del	5.19 Mb	maternal	P	15q11.2q13 BP1-BP3 region	DD, ID, abnormal facies
2	10	F	arr [hg38] 15q11.2q13.1 (22,770,421–28,928,730)×1	del	6.16 Mb	unknown	P	15q11.2q13 BP1-BP3 region	ID, delayed speech and language development
3	3	M	arr [hg38] 15q11.2q13.1 (23,123,715–28,295,199)×1 arr [hg38] 16p13.11 (14,806,185–16,189,012)×3	del dup	5.17 Mb 1.38 Mb	unknown unknown	P P	1.15q11.2q13 BP1-BP3 region 2.16p13.11 BP2-BP3 region	DD, ID, hypotonia
4	1.42	F	seq [hg38] del (15) (q11.2q13.1) chr15:g.23,133,602_28,299,539	del	5.13 Mb	unknown	P	15q11.2q13 BP1-BP3 region	DD, ID, astasia, absent speech, hypertonia
5	5	M	arr [hg38] 22q11.21q11.23 (21,446,182_24,263,610)x1	del	2.82 Mb	maternal	P	22q11.2 distaregion	DD, ID, Cleft lip
6	0.33	M	arr [hg38] 22q11.21 (18,166,088–21,446,182)×1	del	3.15 Mb	unknown	P	22q11.2 proximal region	DD, abnormal facies
7	0.25	M	arr [hg38] 22q11.21 (18,153,983_21,110,475)×1	del	2.96 Mb	unknown	P	22q11.2 proximal region	DD, asymmetric crying facies
8	5	F	arr [hg38] 16p11.2 (29,568,699–30291,027)×3	dup	722 kb	*de novo*	P	16p11.2 proximal region	ID, hyperactivity
9	4.92	M	seq [hg38] del (16) (p11.2) chr16:g.28,823,259_28,990,047	del	167 kb	paternal	P	16p11.2 distal region	DD, ID, absent speech, hydrocephalus; the fathter is unaffected
10	0.58	M	arr [hg38] 7q11.23 (73,309,375–74,739,874)×1	del	1.43 Mb	unknown	P	7q11.23 Williams-Beuren syndrome region	ID, hypospadias
11	1.42	M	seq [hg38] dup(X) (q28) chrX:g.153,830,213_154,329,701	dup	462 kb	maternal	P	Xq28 MECP2 region	DD, ID, hypotonia; The mother is normal
12	1	F	arr [hg38] 5p15.33p14.3 (113,461–22,011,396)×1	del	21.9 Mb	*de novo*	P	Cri du chat syndrome	DD, ID
13	1	F	arr [hg38] 7p22.3p21.3 (43,376–10078044)×3	dup	10.0 Mb	*de novo*	P	7p22.1 region	DD, ID
14	1	F	seq [hg38] del (1) (p36.33p36.31) chr1:g.65489_5,909,266 seq [hg38] dup (21) (q22.3q22.3) chr21:g.43,656,557_46,664,413	del	5.84 Mb 3.01 Mb	*de novo* *de novo*	P	1p36 terminal region	DD, ID, prenatal lateral ventricle dilatation, hearing impariment, choreoathetosis, cerebral hypoplasia
15	0.67	F	seq [hg38] del (22) (q12.3q13.1) chr22:g.36,702,345_38,582,919	del	1.88 Mb	*de novo*	P	*SOX10*	DD, hearing impairment, hypotonia, bilateral malformation of semicircular canal morphology and horizontal semicircular canals, leukodystrophy
16	0.17	M	arr [hg38] 22q12.3q13.1 (37,186,430_38,910,625)×1 arr [hg38] 22q13.31q13.33 (48,016,192_50,759,338)×1	del	1.72 Mb	unknown	P	1. *SOX10* 2. *SHANK3*	DD,ID. hypoplasia of the corpus callosum, hearing impairment
17	3.67	M	arr [hg38]22q13.33 (50,682,948_50,759,338)×1	del	76 kb	*de novo*	P	*SHANK3*	Neurodevelopmental delay, language impairment, autism, abnormal facies (dolichocephaly, bilateral ptosis, wide nasal bridge, pointed chin, large and prominent ears)
18	0.75	M	arr [hg38]7q31.1q31.2 (111,690,830_116,337,226)×1	del	4.65 Mb	*de novo*	P	*FOXP2*	DD, hypotonia, dyspraxia, phonology deficits
19	0.25	M	arr [hg38] Xp21.1p11.4 (31,934,338_39,870,419)×0	del	7.94 Mb	unknown	P	*DMD, CYBB, OTC*	DD, lethargy, respiratory alkalosis
20	0.58	F	arr [hg38] 7q35q36.3 (143,491,502–159,327,017)×1	del	15.9 Mb	*de novo*	P	*KCNH2, KMT2C, SHH, MNX1*	DD, abnormal facies, recurrent fever
21	5	M	arr [hg38] 7q21.13q22.1 (91,364,552_99,779,087)x1	del	8.41 Mb	unknown	P	*SGCE*	DD, ID, hearing impairment, dystonia
22	3.67	M	arr [hg38] 3p23p22.2 (31,194,076_38,634,761)×1	del	7.44 Mb	unknown	P	*MLH1, SCN5A*	DD, short stature, hypotonia, feeding difficulties, depressed nasal bridge
23	5	M	arr [hg38] 2p25.3p11.2 (50,813–241,831,406)×2 hmz	LOH	242 Mb	*de novo*	P	*UNC80*	DD, ID, severe truncal hypotonia, absent speech
24	4.17	M	arr [hg38] 16q24.1q24.2 (86,567,833_88,082,850)×1	del	1.52 Mb	unknown	P	*FOXC2*	DD, ID
25	0.75	M	arr [hg38] 10p15.3p13 (54,108_13,150,380)×1	del	13.1 Mb	unknown	P	*ZMYND11, GATA3*	DD, ID, microcephaly, camptodactyly of toe
26	2	M	seq [hg38] del (20) (p12.3p12.1) chr20:g.8,750,777–13,793,273	del	5.04 Mb	*de novo*	P	*JAG1*	DD, abnormal facies, cholestasis, jaundice, delayed speech and language development, seizures, patent ductus arteriosus
27	7.33	F	arr [hg38]Yq11.222q12 (18,481,471_26,653,507)×0	del	8.17 Mb	unknown	P	AZFb and AZFc region	DD, short stature
28	2.58	M	arr [hg38]13q31.1q34 (87,066,205_114,342,258)×3	dup	27.3 Mb	*de novo*	P	Score 1.50^a^	DD, ID, tetany, language impairment, periventricular leukomalacia
29	0.25	M	arr [hg38] 1q41q44 (223,906,768–248,930,485)×3	dup	25.0 Mb	*de novo*	P	Score 1.20^a^	DD
30	7.58	F	seq [hg38] del (11) (q24.3q25) chr11:g.128,462,360_134,763,813 seq [hg38] dup (21) (q22.12q22.3) chr21:g.34,669,388_46,664,409	del dup	6.30 Mb 12.04 Mb	*de novo* *de novo*	LP P	1. Score 0.90^a^ 2. Score 1.35^a^	DD, ID, abnormal facies (hypertelorism, arched eyebrows), agenesis of corpus callosum, asymmetric growth

Abbreviation: mo, month; y, year; CNV, copy number variant; chr, chromosome; del, deletion; dup, duplication; LOH, loss of heterozygosity; kb, kilobase; Mb, Megabase; P, pathogenic; LP, likely pathogenic; VOUS, variants of uncertain significance; DD, development delay; ID, intellectual disability.

^a^ The detailed criteria and calculations used in determining CNV, scores are provided in Supplementary Table 1.

The 32 clinically significant CNVs included 24 deletions (75.0%), 7 duplications (21.9%), and 1 instance of loss of heterozygosity (LOH) (3.1%).

Of the 43 CNVs identified, inheritance data was available for 21 cases. Fifteen CNVs (65.2%) were *de novo*. Four CNVs were maternally inherited, including two duplications in regions associated with X-linked syndromes. Two CNVs were paternally inherited. The inheritance patterns of the remaining 22 CNVs were unknown, likely due to limited family information or the high cost of parental testing, which some families may not have been able to afford.

### 3.3 High-risk CNV regions and associated phenotypes

Recurrent CNVs are defined as copy number variations that frequently occur at specific genomic regions predisposed to deletions or duplications across individuals (Smajlagić et al., 2021). Among the 33 clinically significant CNVs identified in this cohort, 12 (36.4%) were located in recurrent CNV regions, including 15q11.2-q13.1 (4 cases), 16p11.2 (2 cases), 16p13.11 (1 case), 22q11.2 (3 cases), Xq28 (1 case), and 7q11.23 (1 case). Additionally, *SOX10*-related CNVs were identified in two cases, and *SHANK3*-related CNVs were also found in two cases, with one case presenting deletions in both *SHANK3* and *SOX10*.

### 3.4 Normal CMA results and clinical phenotypes

In this cohort, 59 out of the 99 pediatric patients (59.6%) had normal results, with no clinically significant CNVs detected. Despite the absence of detectable CNVs, these patients exhibited a range of clinical phenotypes, including DD in 36 cases, ID in 25 cases, hypotonia in 9 cases, and delayed speech in 8 cases. Additional clinical features observed included ASD in 5 cases and seizures in 3 cases.

## 4 Discussion

This study investigates the genetic underpinnings of neurodevelopmental disorders through a comprehensive analysis of CNVs in pediatric patients with DD and/or ID. It provides valuable insights into recurrent genomic regions, novel pathogenic variants, and the complexities of inheritance patterns. Recurrent CNVs were identified in 12 cases (36.4%), with 15q11.2-q13.1 being the most frequently observed region (4 cases). This region was predominantly associated with DD (3/4 cases) and ID (3/4 cases), along with variable features such as hypotonia, abnormal facies, and speech delay—phenotypes commonly linked to syndromic conditions like Prader-Willi and Angelman syndromes (Ma et al., 2023). 16p11.2 CNVs, identified in two cases, were associated with ID and behavioral symptoms like hyperactivity and absent speech, aligning with known associations to ASD and ADHD. The observed incomplete penetrance in this region, evidenced by an unaffected parent, underscores the need for family testing, especially with penetrance rates of 33% for distal and 47% for proximal deletions (Goh et al., 2024). This variability is crucial for genetic counseling and reproductive planning, as family testing offers insights into inheritance patterns, allowing for accurate recurrence risk assessment and informed reproductive decisions. 22q11.2 CNVs, observed in three cases, were consistently linked to craniofacial anomalies (e.g., cleft lip, abnormal facies) and DD, findings consistent with DiGeorge syndrome (Szczawińska-Popłonyk et al., 2023). Additional high-risk CNV regions, each identified in one case, included 7q11.23, 16p13.11 and Xq28, each contributing unique but well-documented phenotypic associations in neurodevelopmental presentations. The high recurrence of these CNVs supports the diagnostic value of CMA in neurodevelopmental disorders and further underscores the importance of genetic screening and family testing to clarify inheritance patterns and the variable expressivity of these high-risk regions.

Beyond recurrent CNVs, this study identified unique pathogenic variants affecting genes associated with neurodevelopmental outcomesNotably, deletions related to *SOX10* and *SHANK3* CNVs were each observed in two patients, with one patient exhibiting concurrent deletions of both *SOX10* and *SHANK3*. *SOX10*, which is typically associated with neural crest development and conditions such as Waardenburg syndrome and peripheral neuropathies (Pingault et al., 2022), was found in this study to be linked with DD and ID. This finding suggests broader phenotypic implications that may involve additional genes within the deleted region. In one patient with an isolated *SOX10* deletion, symptoms included hearing impairment, hypotonia, and bilateral semicircular canal malformations, aligning with known *SOX10*-related craniofacial and auditory features. Another patient with an isolated *SHANK3* deletion presented with neurodevelopmental delay, language impairment, autism, and craniofacial features typical of *SHANK3*-associated Phelan-McDermid syndrome (Mitz et al., 2024). In contrast, the patient with combined *SOX10* and *SHANK3* deletions exhibited more complex symptoms, including DD, ID, corpus callosum hypoplasia, and hearing impairment, highlighting potential cumulative effects. This combined deletion profile suggests further investigation into gene interactions and their influence on neurodevelopmental outcomes. Additionally, uniparental disomy (UPD) of chromosome 2 was identified in one patient, attributed to a *UNC80* gene homozygous mutation c.5609-4G>A causing Infantile hypotonia with psychomotor retardation and characteristic facies-2 (Tao et al., 2021). The UPD-associated *UNC80* mutation underscores the critical importance of employing multiple technologies to uncover the genetic basis of “CNV-negative” cases. These findings emphasize the need for a paradigm shift in diagnostic strategies, advocating for the integration of CNV analysis with complementary genomic approaches to better understand the complexities of neurodevelopmental disorders.

The inheritance analysis conducted in this study revealed that 65.2% of CNVs with established inheritance patterns were *de novo*. This finding aligns with prior research that associates *de novo* CNVs with complex phenotypes and heightened risks for developmental disorders (Marrus and Hall, 2017; Brunet et al., 2021). Two CNVs were inherited from the father: one was a distal deletion at 16p11.2 from an unaffected father, and a duplication at 2q12.1q12.3, co-segregating in a father-proband pair exhibiting ID and strabismus. Three additional cases of 2q12.1q12.3 duplications (Kocaay et al., 2022) and two ClinVar/Decipher database entries (Variation ID:152,946; Patient 481,619) consistently report speech delays, cognitive deficits, and motor developmental abnormalities. The 2q12.1q12.3 region of our patients encompasses 30 protein-coding genes, with *POU3F3* emerging as the strongest candidate due to its association with Snijders Blok-Fisher syndrome, characterized by DD, ID, and neurological anomalies. Although *POU3F3* duplications remain unclassified as pathogenic and their molecular mechanisms are undefined, the concordance of neurodevelopmental deficits—such as speech delays, cognitive impairment, and motor dysfunction—across our cases and five previously reported cases strongly suggests a contributory role. However, no established association exists between this duplication and strabismus, highlighting the necessity for further investigation into this relationship. Four CNVs were inherited from the mother, including a case of Xq28 *MECP2* duplication in which the child exhibited DD, ID, and hypotonia, while the mother remained asymptomatic. This finding is consistent with previous studies suggesting that female carriers of *MECP2* duplications or mutations frequently remain unaffected, potentially due to skewed X-chromosome inactivation (XCI), which may diminish phenotypic expression (Pascual-Alonso et al., 2020; Sun et al., 2021). The findings highlight the significance of family-based CNV testing for effective genetic counseling and reproductive planning. The 22 cases of CNVs with unknown inheritance highlight a limitation of this study, likely attributable to the high cost of parental testing, which constrained thorough family analysis for certain patients.

This study identified 11 CNVs classified as VOUS, highlighting the ongoing challenges in interpreting these variants in clinical genetics. The VOUS entries ranged from 288 kb to 6.25 Mb and involved genes associated with autosomal or X-linked recessive disorders, such as PCDH15, GPC3, GPC4, CSF2RA, and CASK, although the observed clinical symptoms may not fully align with these gene associations. Assessing the pathogenic significance of these variants typically necessitates further research and supplementary testing (Jansen et al., 2023). Furthermore, 59.6% of patients presented with normal CMA results while displaying DD and ID, indicating that additional genetic factors—such as single nucleotide variants, epigenetic modifications, or environmental influences—could play a role in the observed phenotypes. Integrating these additional factors in further research is essential for improving diagnostic accuracy and advancing the understanding of the genetic landscape of neurodevelopmental disorders.

Several limitations of our study should be considered when interpreting the results. First, this was a single-center study with a relatively small sample size, which may not fully capture the genetic heterogeneity present in broader populations. Second, the resolution of chromosomal microarray analysis (CMA) employed in this study may not have been sufficient to detect certain classes of small or balanced genomic alterations, including low-level mosaicism or balanced translocations. Third, the interpretation of variants, particularly variants of uncertain significance (VOUS), is constrained by current knowledge and may evolve with advances in genomic databases and annotation tools. Finally, although inheritance was analyzed when parental samples were available, not all CNVs could be definitively classified due to incomplete parental testing.

## 5 Conclusion

This study underscores the utility of CMA and WES as diagnostic tools in pediatric patients with DD and ID, offering insights into the genetic landscape of neurodevelopmental disorders. The findings support CMA as a first-tier diagnostic method and highlight the role of CNV detection for informed clinical decisions and genetic counseling. Limitations include the small cohort size and lack of long-term follow-up, which restrict understanding of the full clinical impact of specific CNVs. Larger, diverse studies with longitudinal tracking are needed to confirm these findings. The interpretation of VOUS remains challenging, and future research using multi-omics approaches, including transcriptomics and proteomics, may help clarify the mechanisms underlying VOUS and CNV variability.

## Data Availability

The dataset associated with our manuscript has been deposited in the China National GeneBank Database (CNGBdb) under the accession numbers CNP0007454 and CVAR0000372. Interested researchers may request access by contacting CNGBdb and citing the accession numbers.
